# Adherence to anti-retroviral therapy among HIV patients in Bangalore, India

**DOI:** 10.1186/1742-6405-6-7

**Published:** 2009-04-28

**Authors:** Mary B Cauldbeck, Catherine O'Connor, Mortimer B O'Connor, Jean A Saunders, Bhimasena Rao, V G Mallesh, Nagendrappa Kotehalappa Praveen Kumar, Gurushanthappa Mamtha, Claire McGoldrick, Robert BS Laing, Kadappa Shivappa Satish

**Affiliations:** 1 The School of Medicine, University of Aberdeen, Aberdeen, Scotland, UK; 2 GU/STD Clinics, Department of GU/STD Medicine, Mid-Western Regional Hospital, Limerick, Ireland; 3 Department of Medicine, South Infirmary – Victoria University Hospital, Cork, Ireland; 4 The School of Medicine, University College Cork, Cork, Ireland; 5 Department of Mathematics and Statistics, University of Limerick, Limerick, Ireland; 6 Department of Respiratory and HIV Medicine, Rajajinagar and Wockhardt Hospital and Heart Institute, Bangalore, India; 7 Infection Unit, Department of Infectious Diseases, Aberdeen Royal Infirmary, Aberdeen, Scotland, UK

## Abstract

**Introduction:**

*Human Immunodeficiency Virus *(HIV) has an estimated prevalence of 0.9% in India (5.2 million). Anti-retroviral drugs (ARV) are the treatments of choice and non-adherence is an important factor in treatment failure and development of resistance, as well as being a powerful predictor of survival. This study assesses adherence to ARV in HIV positive patients in Bangalore, India, a country where only 10% of those who need therapy are receiving it.

**Methods:**

A cross-sectional anonymous questionnaire survey of 60 HIV antibody positive patients was carried out with patients attending HIV outpatient services at two centres: The Chest and Maternity Centre, Rajajinagar, and Wockhardt Hospital and Heart Institute, Bangalore. Consent was obtained. Translation was done by a translator and doctors where required. Data was analysed using SPSS statistical analysis.

**Results:**

A response rate of 88% (53/60) was achieved. The mean patient age was 39.98 years, with 50% aged 30–40, and 73.6% of participants being male. Mean family size was 4.8 (1–13). 21% lived less than 50 kms and 21% greater than 400 kms from clinic.

60% reported they were fully adherent. Adherence was statistically significantly linked to regular follow-up attendance (70.5%, p = 0.002). No other results were statistically significant but trends were found. "100% adherence" trends were seen in older patients, male gender, those from larger families, those who had a previous AIDS defining illness, those taking fewer tablets, and without food restrictions. Commonest side-effects causing non-adherence were metabolic reasons (66%) and GI symptoms (50%). No trends were seen for education level, family income, distance travelled to clinic, time since diagnosis, or time on ART.

**Conclusion:**

Regular attendance for follow up was statistically significant for 100% lifetime adherence. Positive trends were seen in those in larger families, older, those who had AIDS defining illness, simple regimes, and without side-effects. Education, income, distance travelled and length of time diagnosed or treated had no effect on adherence.

## Introduction

*Human Immunodeficiency Virus *(HIV) affects 40.3 million people worldwide [1]. Sub-Saharan Africa, with an estimated 25.8 million carriers, has the greatest burden of disease (64%) [1,2]. The prevalence is estimated at 5–15% in most other Sub-Saharan countries [1,2]. Asia has an estimated prevalence in the region of 8.3 million, with 5.2 million being in India, giving an Indian adult HIV prevalence of 0.9% [3]. This compares to an estimated 58,300 cases in the United Kingdom (UK) at the end of 2004 [4]. In India, HIV prevalence tends to be higher in industrialized states including Karnataka [1], with infection levels of over 1% found in pregnant women in Andhra Pradesh, Karnataka and Maharashtra in 2004 [1].

Worldwide, the major route of transmission is unprotected heterosexual intercourse (>75%) [5,6]. This accounts for the increasing number of women being affected worldwide, including India. Homosexual sexual intercourse is the second commonest route of transmission, with the exception of localised areas such as Sydney, Australia [7]. The incidence in injection drug-users (IDU) varies internationally. It is relatively low in the UK [8] while it is up to 50% in Eastern Europe, Vietnam, northeast India and China [2]. Eastern Europe has the fastest growing IDU HIV positive incidence in the world at present [1]. Unfortunately, approximately 5–10% of new HIV infections, worldwide, are in children; >90% of these are infected during pregnancy, birth or through breast-feeding [2,9]. Most of these cases occur in Africa, the Caribbean or South East Asia [2,9].

Combination therapies of anti-retroviral drugs (ARV) are the treatment of choice in HIV, and non-adherence is a main, if not the most important factor in treatment failure, and the development of resistance [10]. To make sure that HIV treatment works effectively it is important that the drugs are taken on time, every time i.e. 100% medication adherence. It can be hard to achieve 100% medication adherence, but Sethi AK and colleagues have previously shown that it is "all or nothing" [11]. This study showed that if medication adherence levels were between 70% and 90% then there was an increased risk of resistance forming [11]. In fact it would be better to take no drugs at all than to have adherence of less than 90% [11]. If 95% medication adherence is not achieved then treatment success becomes precarious [12]. It has also been demonstrated that a 10% higher level of adherence results in a 21% reduction in disease progression [13]. These are founded on "Medication adherence" meaning all drugs are taken on time, every time as prescribed by the prescribing physician.

Concerns about incomplete medication adherence among patients living in poverty have been an important consideration in expanding the access to anti-retroviral therapy (ART) [14]. The research into medication adherence in poorer communities however tends to be centred on research undertaken in sub-Saharan Africa. There is a paucity of medical literature on adherence to ART in Asia, however a study has been undertaken in Chennai, India (n = 304) [15]. This study reported patient medication adherence as "regular" in 74.3% of patients, with a significant minority being categorised as "irregular" (17.8%), or "recently missed doses" (6.9%) [15]. Medication adherence was not associated with any demographic variables [15]. In Asia the number of people receiving ART rose from 70,000 in 2003 to 180,000 at the end of 2005, however still only 1 in 6 people in need of ART in Asia are receiving it and coverage is well below 10% in India (which has more than 70% of Asia's total treatment needs) [3]. With a growing prevalence of HIV across all areas of Indian society there is a growing number needing treatment and greater strain on services to provide ART. This in turn means a greater number who potentially have medication adherence issues and questions what facilitates medication adherence from an individual and public health perspective. Some people debate that in treatment stretched areas 100% medication adherent patients should be treated over non-adherent patients.

In view of the high prevalence of HIV in India and the lack of data surrounding medication adherence in this population, it was decided to study adherence to ART issues among a HIV positive population in Bangalore, India. The aims of this study were to evaluate the adherence to ART among HIV patients attending Out Patient clinics at The Chest and Maternity Centre, Rajajinagar, and Wockhardt Hospital and Heart Institute in Bangalore, India. Patients were evaluated by eliciting demographics, treatment regimens and side effects as well as subjective medication adherence.

## Methods

HIV antibody positive patients attending out-patient clinics in two institutions, The Chest and Maternity Centre, Rajajinagar, and The Wockhardt Hospital and Heart Institute, Bangalore, India were studied.

Participants were recruited intermittently but consecutively while researchers and translators were in attendance over a seven week period between 28^th ^August and 30^th ^September 2006. Both researchers and a translators were required to be present for patient participation in the study to achieve complete patient understanding and avoid any misinterpretations. All patients presenting to clinics, while researchers and translators were present, were eligible for inclusion in the study and therefore approached to participate. No incentives were offered to patients approached for inclusion to limit any bias.

A cross-sectional self-administered anonymous questionnaire survey was administered to HIV antibody positive patients on ART attending the Out Patient services at the two centres. Patients themselves completed a paper format questionnaire, which was explained in detail prior to completion. Each question in the 32-point questionnaire was explained before completing the questionnaire and a translator was present when the patients were completing questionnaires to resolve any questions regarding the questionnaire. Each of the 32-point questions were tick box in format with area for written expansions where relevant. A target of 60 patients to be studied was set.

Informed consent was obtained, and the number of patients refusing recorded.

Questionnaires elicited patient demographics, treatment regimens and side effects as well as subjective medication adherence. Medication adherence was recorded as taking all medications as prescribed by the prescribing physician (All medications at correct time on correct day). Last week, last month, last six month and lifetime adherence was recorded.

Data was analysed using the statistical package SPSS. Chi-square tests, Student's *t *tests, significance testing, and 95% Confidence Interval formulation were carried out where appropriate. Ethical approvals were prospectively received in both centres.

Prior to commencement of the study a one-week pilot study in the two units was undertaken to look for flaws in the questionnaire.

## Results

The response rate was 88% (53/60 agreeing to be involved). Of the responding 53 patients 16 (30%) refused to answer questions about individual and/or family income. These patients were then omitted from statistical analysis on the effect of income on medication adherence.

The mean age was 39.98 ± 2 years, with males being older (40.35 (range: 28–66 years) versus 38.4 (range: 27–60 years)), and 50% of participants aged between 30 and 40 years. 73.6% (n = 39) of participants were male and 26.4% (n = 14) female, with the mean family size of 4.8 (range: 1 – 13). There was a predominance of patients from urban locations compared to rural locations (44 (83%) versus 9 (17%)), while 10 (21%) patients lived less than 50 km from clinic, 8 (17%) 50 – 200 km, 20 (42%) 200 – 400 km, and 10 (21%) greater than 400 km form clinic.

19% of patients missed their medications in the last week, 30% in the last month, 36% in the last six months, and 40% since commencing treatment, in a setting of a mean time on ART of 2.75 years. Medication adherence was recorded as taking all medications everyday as prescribed. Due to difficulties in translation of the meaning of percentage (eg 90%), a mean and median time of 2.75 years on ART for the cohort, no resistance testing available in the centres, along with studies showing that >90% – 95% medication adherence is required in successful HIV treatment/resistance prevention, medication adherence for analysis was taken as lifetime adherence with the gold standard as 100% medication adherence since starting ART.

Medication adherence was analysed under the areas of: 1 basic demographics, 2 system, health and treatment factors, and 3 study factors.

It was recorded on data analysis that regular clinic follow up was the only statistical factor affecting medication adherence (last week (p < 0.005), last month (p < 0.005), last six month (p < 0.005) and lifetime (p = 0.002)) in this cohort. Positive trends for medication adherence were seen for increasing age, larger families, having previously had an AIDS defining illness, smaller pill burden and less medication side-effects experienced.

Table 1 and table 2 outline the principle findings of the study.

**Table 1:** Patient Demographics relative to adherence of medication regimens

	**Non adherent**	100% Adherent *
Age Group (years)		
• 20–30	3 (42.9%)	4 (57.1%)
• 31–40	14 (53.8%)	12 (46.2%)
• >40	4 (23.1%)	16 (76.9%)

Gender		
• Male	15 (38.5%)	24 (61.5%)
• Female	6 (42.9%)	8 (57.1%)

Follow-up		
• Regular #	13 (29.5%)	31 (70.5%)
• Irregular	8 (88.9%)	1 (11.1%)

Education Level		
• Illiterate	2 (50%)	2 (50%)
• Primary/Secondary	9 (80%)	18 (20%)
• University	10 (45.5%)	12 (54.5%)

Individual Income (Rs/month)		
• <5000	5 (45.5%)	6 (54.5%)
• 5000–19999	8 (27.3%)	15 (72.7%)
• >20000	7 (43.8%)	9 (56.3%)

Total Family Income (Rs/month)		
• <5000	2 (33.3%)	4 (66.7%)
• 5000–19999	8 (50%)	13 (50%)
• >20000	8 (50%)	8 (50%)

Family Type		
• Living alone	1 (100%)	0
• Nuclear	14 (41.2%)	20 (58.8%)
• Joint/Extended	6 (37.5%)	12 (62.5%)

Locality		
• Rural	3 (33.3%)	6 (66.7%)
• Urban	18 (40.9%)	26 (59.1%)

Distance from Clinic (Km)		
• <50	4 (33.3%)	6 (66.7%)
• 50–200	3 (37.5%)	5 (62.5%)
• 200–400	7 (25.0%)	13 (75.0%)
• >400	3 (30%)	7 (70.0%)

* Lifetime Adherence means taking all medications as prescribed since starting treatment.

# Regular follow up meaning more than 90% attendance at 3 monthly appointments

**Table 2:** Patient Medication adherence

Missed Medications in Lifetime*	**Yes** **n – value (%)**	**No** **n – value (%)**
Ever Missed medications since starting ART^$^	21 (39.6%)	32 (60.4%)

Missed medications^$ ^in the last 6 months	19 (35.8%)	34 (64.2%)
Missed medications^$ ^in the last month	16 (30.2%)	37 (69.8%)
Missed medications^$ ^in the last week	10 (18.9%)	43 (81.1%)

Patient perception of well-being		
• Sick	3 (50.0%)	3 (50%)
• Stable	9 (39.1%)	14 (60.9%)
• Normal	9 (37.5%)	15 (62.5%)

Previous AIDS defining illness:		
• Yes	9 (28%)	24 (72%)
• No	10 (50%)	10 (50%)

Ailments presently suffering from		
• AIDS	8 (27.6%)	21 (72.4%)
• Well(none)	4 (44.4%)	5 (55.6%)
• Ill not AIDS	9 (60%)	6 (40%)

Time since Diagnosis (years)		
• <1	2 (33.3%)	8 (66.7%)
• 1–4	8 (44.4%)	10 (55.6%)
• 5+	13 (35%)	14 (65%)

Number of years since patient became ill		
• < 1 yr	5 (57.1%)	8 (42.9%)
• 1–4	9 (39.1%)	14 (60.9%)
• 5+	7 (35.7%)	10 (64.3%)

Time since starting ART (years)		
• < 1	5 (50%)	8 (50%)
• 1–4	10 (35.7%)	18 (64.3%)
• 5+	6 (50%)	6 (50%)

Number of tablets/day		
• <5	7 (24.1%)	22 (75.9%)
• 5–9	10 (58.8%)	7 (41.2%)
• 10+	3 (50%)	3 (50%)

Food Restriction		
• Yes	12 (52.2%)	11 (47.8%)
• No	9 (30%)	21 (70%)

Temperature Restriction		
• Yes	3 (60%)	2 (40%)
• No	18 (37.5%)	30 (62.5%)

Time Restriction		
• Yes	12 (50%)	12 (50%)
• No	9 (31%)	20 (69%)

Perceived difficulty of regimen		
• Simple	11 (33.3%)	22 (66.7%)
• Moderate	10 (50%)	10 (50%)

Any Side Effects		
• None	9 (34.6%)	17 (65.4%)
• GI symptoms^@^	6 (50%)	6 (50%)
• CNS	2 (40%)	3 (60%)
• Rash/Skin discolouration	0	3 (100%)
• Metabolic reasons (non-lipid)^^^	2 (66.7%)	1 (33.3%)
• Lipid Problems	2 (50%)	2 (50%)

* Lifetime Adherence means taking all medications as prescribed since starting treatment.

^$ ^Missed medications means not taking a prescribed medication on any day as prescribed by the physician.

^@ ^GI symptoms = Gastrointestinal tract symptoms which include diarrhoea, nausea and vomiting.

^^ ^Metabolic reasons include diabetes mellitus.

## Discussion

Overall 100% medication adherence was found to be 60.4% (n = 32), p-valve = 0.615. This was lower than expected as other studies conducted in developing countries have shown a "regular" medication adherence of 74.3% [16]. Of the 39.6% who had missed a medication, approximately 50% had missed a medication in the last week, and 40% had missed because they had run out of medications. Missed medications means not taking a prescribed medication on any day as prescribed by the physician. Of the patients who had run out of tablets all lived more than 250 km from the clinic. This may imply that there is a lack of availability of medicines in some geographical areas. It is difficult to understand why medication adherence should be less in this sample although it may have something to do with the criteria for measuring medication adherence. During data collection for this study only those with 100% medication adherence were considered as adherent whereas in other studies the level of medication adherence is calculated and a level above 95% is taken to be adherent i.e. missing one medication in twenty is acceptable.

Older patients showed a tendency towards better medication adherence but not statistically significant (p = 0.325). This may be related to older patients' familiarity with medication usage and their increasing awareness of HIV as a disease that requires optimal adherence [17]. It was those aged less than 40 years in this study that showed the poorest levels of medication adherence. Many other studies have also identified young age as a risk factor for poor medication adherence especially in those under 35 years [16,18].

Gender was not associated with medication adherence, in our study, as is supported in a previous review article of 18 descriptive studies [19]. Surprisingly neither living in a rural compared with an urban area nor travelling long distances to the clinic showed trends against medication adherence (p = 0.479). It is difficult to draw conclusions related to gender as there are few female in this study.

It has been shown in this study that attending for regular follow-up with a doctor correlates with better medication adherence (p = 0.002). Regular follow up being defined as attending greater than 90% of 3 monthly appointments. This may be due to continued counselling, patients being able to express concerns about their health and medications, and receiving supplies their medications avoiding running out.

Surprisingly literacy was not significantly associated with lower medication adherence (p = 0.089). This has also been shown by Cheng et al in a 2006 publication [20]. Verbal instructions to patients who are illiterate seem equally as effective as written instructions which are given to all patients.

One may assume that those of a higher social status and income are more adherent to their ART. This was not seen here, where we report neither a patient's individual income (p = 0.786) nor their total family income (p = 0.9) show any significant association regards 100% medication adherence. This was unexpected, as patients in India must pay for their medications. A study undertaken in Chennai, India found that almost all the participants discussed the cost of ART as a barrier, with many reporting drug holidays, turning to family and/or friends or taking drastic measures (i.e. selling family jewels, property) for financial assistance [21]. It can be seen however that those in the middle class earning between 5000–9999 Rs/month (Approximately GB£60–125) seem to be better at adhering with medications compared to the very rich and very poor who seem to be less careful. It must be questioned if many of those who earned less than 5000 Rs/month could actually afford to buy their medicines.

Due to the small sample size of this cohort it is difficult to reach valid conclusions on whether family size or type is associated with medication adherence. This question was originally posed in order to determine whether lack of privacy within the household affected patient medication adherence, as this is a known effect. On reflection, it would have also been important to ask if other members of the household knew of the patients HIV status in order to analyse this fully. Looking at our results, however, trends were seen, despite not significant, as medication adherence was better with increasing numbers of family members. This may be due to the fact that patient's family members may be useful to remind patients to take their tablets regularly, or to help to pay for medicines, or both. Poorer medication adherence was also seen in those living alone (p = 0.407). It may therefore be argued that increased acceptance and understanding of the disease, thus reducing social stigma, and knowledge of the disease treatments may improve medication adherence.

Results did show a trend towards an increased reporting of 100% medication adherence in those interviewed with a doctor as a translator. A study conducted by Hautzinger M et al in 2006 showed that patients are more likely to inform their doctor why they take, than why they do not take, ART [22]. Only half of those who were non-adherent told their physician the reason why they missed medications [22]. The reasons for not telling their physician included feeling unable to discuss issues and anticipation that the doctor would not support the decision [22]. Therefore using a doctor involved in a patient treatments as a translator in this study and future studies may bias results and should be avoided in the future.

Although patient perception of their own physical well-being did not affect medication adherence statistically, it was seen that 50% of those who reported themselves as feeling 'sick' admitted to having missed medications in the past. This supports the fact that missing more than 5% of ART medications can lead to a more rapid decline into ill health [14]. Of those who were suffering from an AIDS defining illness at the time of the study, they were less likely to skip tablets as they improved their health.

Being on ART for less than 6 months or for greater than 10 years was associated with the largest number of missed medications, in this study. This finding is supported by Andreo C. et al who found that a duration of treatment greater than two years was associated with increased non-adherence to ART [23]. Tablet and schedule burdens can often, understandably, be assumed to affect any medication adherence. In this Indian cohort we saw a trend towards increased 100% medication adherence in those on less than 5 tablets per day (p = 0.054). The number of times per day that tablets were taken was also analysed but this yielded no associations. This may be because most patients (37 out of 53) are taking once or twice a day regimens as they have started their ART since the advent of Highly Active ART. Older studies of medications adherence showed that once daily or twice daily dosing would give 95% medication adherence while three times per day scheduling reduced it to 60% [24]. Food restrictions (p = 0.157), time restrictions (p = 0.259) and temperature restrictions (p = 0.374) seemed to decrease medication adherence as was seen in other studies [24], while those that experience side-effects from their medications are known to be 'risk patients' for non-adherence to ART [14], as was shown in this study. However, the severity of the side effects also seemed to be a factor. Those experiencing milder side effects such as skin rash or skin discolouration were more adherent to ART than those experiencing more severe side effects such as metabolic effects.

From the findings of this study, we noted that patient's holding their own notes makes follow up by professionals difficult in out patient's clinic setting along with affecting 100% medication adherence. Therefore we recommend the appointment of a person(s) to remind patients of their regimens and recall them to clinic when arranged. The use of mobile phones, which are becoming universally available, are a big help to communicate with patients directly. A text or a call may be all that is necessary to achieve a greater number achieving 100% medication adherence by attending when arranged. The use of pill boxes, which are filled weekly, may also be beneficial as they enable patients to know with certainty that they have taken medicines.

Factors affecting lifetime medication adherence correlate with factors affecting 1 week adherence, 1 month adherence and 6 months adherence.

## Conclusion

More people who do not attend regularly are less adherent to ART from a 100% medication adherence perspective then their dedicated counterparts. Greater medication adherence was also seen in those who were older, male gender, from larger families, have a previous AIDS defining illness, and taking less tablets in a day (especially <5 tabs). Patients without food, temperature or strict time restrictions also did better.

There was no trend in 100% medication adherence when comparing educational status, distance living from clinic and length of time since diagnosis. There was, however, a trend of non-adherence in those who were on ART for longer (especially those >10 years experience).

This study shows that many patients in India are getting ART but many have to travel long distances for it and have to pay for medication. CD4+ analysis is easily available but viral loads are not as freely available, and all tests have a cost to the patient.

Further larger study into medication adherence among Indian HIV patients is required, especially looking at the impact of income and medication adherence. Future studies should also look at adherence on shorter time scales, for adherence among Indian HIV patients, rather than lifetime adherence as in this study.

## Competing interests

The authors declare that they have no competing interests.

## Authors' contributions

MC, BR, GM, KP, DM, KS carried out the field work. COC, MOC, JS carried out the statistical analysis. MC, COC, MOC prepared the manuscript. MC, CM created the database. MC, COC prepared the study design. RL, KS were guarantors of the study.
